# Integrated primary care: patient perceptions and the role of mental health stigma

**DOI:** 10.1017/S1463423618000403

**Published:** 2018-06-19

**Authors:** Lisa R. Miller-Matero, Shehryar Khan, Rachel Thiem, Tiffany DeHondt, Hala Dubaybo, Daniel Moore

**Affiliations:** 1 Department of Behavioral Health, Henry Ford Health System, Detroit, MI, USA; 2 Department of Internal Medicine, Henry Ford Health System, Detroit, MI, USA; 3 Center for Health Policy & Health Services Research, Henry Ford Health System, Detroit, MI, USA

**Keywords:** integrated primary care, mental health stigma, patient perceptions

## Abstract

Some patients are more willing to see a behavioral health provider within primary care. The purpose of this study was to evaluate the patients’ perspectives of having access to a psychologist within primary care and to investigate whether mental health stigma affected preferences. In total, 36 patients completed questionnaires after seeing a psychologist in primary care. Patients were satisfied with having a primary care psychologist involved in their care. Most patients were more likely to see the psychologist in primary care and those who preferred this indicated higher levels of mental health stigma. The overarching theme for why patients saw a psychologist in primary care was convenience. Mental health stigma may also have played a role. Results suggest that providing integrated services may reach patients who may not have otherwise sought services in a behavioral health clinic. Findings from this study encourage the continued integration of behavioral health services.

## Introduction

Approximately 30% of adults with a physical health disorder have one or more behavioral health conditions, such as depression, anxiety, and substance use disorders (Substance Abuse and Mental Health Services Administration, 2012; Clement *et al*., 2015). Many patients with behavioral health problems are seen by primary care providers (Butler *et al*., 2008; Crowley and Kirschner, 2015); however, very few patients referred to behavioral health by their primary care providers will follow through with the referral (Collins and Fund, 2010). One promising strategy to improve care is to integrate behavioral health services into the primary care clinic (Ede *et al*., 2015).

There are many reasons hypothesized for why patients are more willing to see a psychologist in primary care than a behavioral health clinic. High levels of patient satisfaction have been demonstrated with integration of behavioral health services (Pomerantz *et al*., 2008; Funderburk *et al*., 2010; Reid *et al*., 2010; Schoen *et al*., 2011; Jackson *et al*., 2013; Reiss-Brennan, 2014; Crowley and Kirschner, 2015). Patients report having better access to care (Reid *et al*., 2010), perceive their care as better coordinated (Reid *et al*., 2010; Schoen *et al*., 2011; Jackson *et al*., 2013), and feel as though they are being treated from a more holistic perspective (Funderburk *et al*., 2010; Reiss-Brennan, 2014). Mental health stigma has been found to be a major deterrent to seeking behavioral health treatment (Butler *et al*., 2008; Corrigan, 2004; Corrigan *et al*., 2014). Although it is hypothesized that receiving psychiatric treatment in primary care setting may reduce stigma associated with going to a mental health clinic, this has not yet been directly evaluated. Additionally, other reasons for patients’ preferences of receiving behavioral health treatment in primary care have not yet been well researched.

The purpose of this study was to evaluate the patients’ perspectives of having access to a psychologist within a primary care clinic. The aims were to determine the reasons that patients chose to see an integrated primary care psychologist, to evaluate their satisfaction with this service, and to investigate whether mental health stigma affected patient preferences.

## Methods

### Participants

In total, 36 patients who were seen by a psychologist and/or psychology intern in an Internal Medicine primary care clinic at an urban hospital between January and June 2015 completed brief questionnaires. The majority of participants were female (66.7%, *n*=24). Most patients identified as Black or African-American (75%, *n*=27), 22.2% (*n*=8) identified as White or Caucasian, and 2.8% (*n*=1) identified as biracial.

### Materials

Participants were asked to report on the reasons they chose to see the psychologist in the primary care clinic. They were provided with a list of possible options (Table 1) and were instructed to choose up to three responses for the top reasons. They also had the option to write in additional responses. Participants were also asked their location preferences for behavioral health treatment (e.g., being seen in a primary care clinic, behavioral health clinic, or no preference). If patients did not want to see a psychologist in the primary care clinic, they had the option to see another mental health provider in a behavioral health clinic.

**Table 1:** Patients’ main reasons they chose to see the primary care psychologist

Reason	%	*n*
The physician recommended it	69.4	25
I wanted help	38.9	14
I could be seen the same day	33.3	12
I was seen in the primary care clinic	33.3	12
The psychologist worked directly with the physician	33.3	12
It was easy to schedule an appointment	25.0	9
It was convenient	19.4	7
It was less embarrassing than going to a Behavioral Health Clinic	11.1	4

Participants completed a brief measure on satisfaction with integrated care services (Table 2). Responses were on a five-point Likert scale from strongly disagree to strongly agree. Although this is not a validated measure, a similar version has been used to assess patient satisfaction with integrated psychology services in an oncology department (Jesse *et al*., 2015).

**Table 2:** Patient satisfaction with services

Question	%^a^	*n*
Having the psychologist involved in my care was helpful	90.3	28
My ability to cope with my medical situation improved because I saw the psychologist	71.4	20
The psychologist helped reduce my level of distress	69.0	20
The psychologist helped improve communication between me and my medical providers	70.8	17
My experience as a patient was better when the psychologist was involved	96.2	25
Meeting with the psychologist improved my experience with the Internal Medicine clinic	76.9	20
I appreciated having a psychologist available to me in Internal Medicine	93.8	30
I am grateful that a psychologist was involved in my care	93.8	30

a Percentage of those who agreed or strongly agreed with each of the statements.

The Disclosure subscale of the Stigma scale, a validated measure, was used to measure mental health stigma (King *et al*., 2007). This subscale consisted of 10 items on a five-point Likert scale (e.g., strongly disagree to strongly agree). Higher scores indicate higher levels of mental health stigma. Internal consistency of this subscale was 0.85.

### Procedure

Participants were recruited from an Academic Internal Medicine Primary Care Clinic in an urban setting. The ‘warm handoff’ model is utilized in this clinic where the primary care provider introduces the idea of the psychology team to patients that he/she believes would benefit from services. The primary care provider then discusses the reason for referral with the psychology team and a member from the psychology team would introduce themselves to the patient while the patient is still in the clinic. There was one staff psychologist and one psychology intern who were available to see patients in this clinic. The initial evaluation included a semi-structured diagnostic interview and often included several screening measures to assess for cognitive functioning, psychiatric symptoms, and health literacy. Brief interventions were provided to the patients (i.e., psychoeducation, diaphragmatic breathing, and behavioral activation).

After patients completed interventions, they were invited to complete the questionnaires described above. All patients who were seen over a three-month period were contacted by mail and invited to participate. The questionnaires were mailed to the patients’ homes and patients were provided with a self-addressed, postage paid envelope to return their questionnaires. This study is approved by the institution’s IRB (IRB no. 9086).

Analyses were conducted with SPSS version 22. Percentages were calculated for reasons endorsed for seeing the integrated care psychologist, location preference for behavioral services, and satisfaction items. An independent samples *t*-test was conducted to determine where there were differences in mental health stigma scores between those who preferred being seen by a behavioral health provider in a primary clinic and those who either preferred a behavioral health clinic or did not have a location preference.

## Results

### Reasons for seeing the integrated behavioral health provider

There were a variety of reasons endorsed for why patients chose to see a psychologist integrated into a primary care clinic (Table 1). The most frequently endorsed reasons included: (1) because their physician recommended it, (2) they wanted help, (3) they could be seen the same day, (4) they were able to be seen in the primary care clinic, and (5) they liked that the psychologist worked directly with their physician.

### Satisfaction with integrated care

The overwhelming majority of patients were satisfied with having a primary care psychologist involved in their care (Table 2). Patients thought the involvement was helpful, it reduced their distress, and they were grateful that a psychologist was involved with their care.

### Role of mental health stigma

Most patients reported they were more likely to see the psychologist in a primary care clinic than at a behavioral health clinic (75.0%, *n*=24). Over one-third of patients reported they would only see a psychologist if it was in a primary care clinic (37.1%, *n*=13).

Scores on the stigma measure varied widely (0–39), with a mean of 19.68 (SD=9.48). Those who preferred seeing a psychologist in a primary care clinic endorsed higher levels of mental health stigma (M=22.40, SD=12.07) compared with those who preferred a behavioral health clinic or did not have a preference (M=10.00, SD=5.70; *t*=2.15, *P*=0.05).

## Discussion

Patients reported multiple reasons for choosing to see an integrated behavioral health professional in a primary care clinic as opposed to seeking treatment at a behavioral health clinic. Although the most commonly rated reason for choosing to see the integrated psychologist was a recommendation from the physician, very few patients follow through with a referral to a behavioral health clinic (Collins and Fund, 2010). There is a higher rate of patients who follow through with a referral when a psychologist is integrated in a primary clinic, suggesting that there are additional factors influencing the decision (Miller-Matero *et al*., 2015). One overarching theme among patients in the current sample appeared to be convenience. Previous research suggests factors affecting behavioral health appointment attendance includes convenience and accessibility (Ede *et al*., 2015). Patients who present with a mental health concern and comorbid health problems can wait weeks to months to be seen by a mental health provider in a specialty behavioral health clinic (Ede *et al*., 2015). With the incorporation of an integrated psychologist on site, the average wait time in this clinic was reduced to the same day or within days.

Patients in this study were also overwhelming satisfied with the integrated care services, and also reported improvement in their distress, which is consistent with previous research (Pomerantz *et al*., 2008; Funderburk *et al*., 2010; Reid *et al*., 2010; Schoen *et al*., 2011; Goodrich *et al*., 2013; Jackson *et al*., 2013; Reiss-Brennan, 2014; Crowley and Kirschner, 2015). The current study also found additional reasons patients were satisfied with these services, including a better experience with the primary care clinic and improvement in communication with their primary care physician. It appears that many patients prefer their primary care provider to have communication with a behavioral health provider; however these providers often do not have regular communication when located at different sites (Blount, 2003). In fact, many primary care providers often do not develop relationships with mental health providers until they felt like the patient is doing poorly (McGough *et al*., 2016). It is likely easier for providers to have regular communication about patients when they are located in the same clinic.

Participants who reported higher levels of mental health stigma preferred receiving behavioral health services in a primary care setting. Many patients want to conceal their mental health problems from others (Brain *et al*., 2014) and may feel that being seen in a primary care clinic would mean it is less likely to be discovered that they are getting treatment for mental health. Providing integrated services in primary care may reach patients who may not have otherwise sought services in a behavioral health clinic due to higher levels of mental health stigma. Patients who are able to overcome the stigma of an initial visit to a behavioral health provider in a primary care clinic may be more likely to follow up with treatment (Miller-Matero *et al*., 2015). Findings from this study provide further evidence of possible benefits of integrated care and encourage the continued integration of behavioral health services into primary care.

There were several limitations with this study. First, only patients who followed through with the referral to see the integrated care psychologist were invited to participate. Those who were recommended, but did not see the psychologist, may vary with regard to mental health stigma and their reasons for pursuing or not pursuing care. It may be useful to evaluate these patients with regards to mental health stigma and other barriers to accessing behavioral health services. Another limitation was that there was a small sample size and data was only collected within an urban setting. It is possible that mental health stigma may vary in different geographical locations. Finally, findings should be interpreted with caution given that the patients who chose to see the psychologist are also likely those who would be satisfied with this type of service.

There are a variety of reasons that may prevent patients from seeking help from behavioral health providers, including difficulty accessing behavioral health providers and a high level of mental health stigma. Findings from this study provide preliminary evidence that offering integrated behavioral health services in primary care could increase utilization among those with higher levels of mental health stigma.
